# Viscoelastic Hydrogel Promotes Disc Mechanical Homeostasis Repair and Delays Intervertebral Disc Degeneration via the Yes-Associated Protein Pathway

**DOI:** 10.34133/bmr.0150

**Published:** 2025-03-04

**Authors:** Zilin Yu, Kang Wu, Chunyang Fan, Jiale Wang, Fengcheng Chu, Wei He, Zhongwei Ji, Yongkang Deng, Di Hua, Yao Zhang, Dechun Geng, Xiexing Wu, Haiqing Mao

**Affiliations:** ^1^ Department of Orthopaedic Surgery, Orthopaedic Institute, The First Affiliated Hospital, Suzhou Medical College, Soochow University, Suzhou, Jiangsu, China.; ^2^ Department of Orthopedics, Wuxi Ninth People’s Hospital Affiliated to Soochow University, Wuxi, Jiangsu, China.; ^3^ State Key Laboratory of Molecular Engineering of Polymers, Department of Macromolecular Science, Fudan University, Shanghai 200433, China.; ^4^ Department of Orthopaedic Surgery, Zhangjiagang Hospital affiliated to Soochow University, Suzhou, Jiangsu, China.; ^5^ Center for Rehabilitation Medicine, Department of Pain Management, Zhejiang Provincial People’s Hospital, Affiliated People’s Hospital, Hangzhou Medical College, Hangzhou, Zhejiang, China.; ^6^ Department of Medical Oncology, The First Affiliated Hospital of Soochow University, Suzhou 215006, China.

## Abstract

Intervertebral disc degeneration (IDD) process is accompanied by overactive inflammation and mechanical instability of the nucleus pulposus (NP). Current treatments do not fully restore the biomechanical environment of discs, limiting their therapeutic efficacy. Thus, novel strategies are required to combat IDD. Hydrogels have outstanding biocompatibility and mechanical properties, most importantly, absorbing and retaining water similar to human NP tissue, showing a unique superiority in the treatment of IDD. In this study, we employed a viscoelastic ionic hydrogel (VIG) composed of polyvinyl alcohol and magnesium ions to investigate the therapeutic effect for IDD. VIG demonstrated an optimal degradation rate and NP-biomimetic swelling behavior in vitro. In the rat model of IDD, VIG-injected discs demonstrated mechanical properties approximating those of native discs, including stiffness, relaxation, and dissipation capacity. Furthermore, finite element analysis demonstrated that VIG improved biomechanical function of degenerated discs. VIG effectively inhibited the progression of IDD in the rat model by increasing extracellular matrix synthesis and decreasing matrix metalloproteinase-13 (MMP-13) expression. Moreover, VIG promoted proliferation and differentiation of NP cells in response to extracellular mechanical changes through the integrin–YAP signaling pathway. These findings suggest that VIG has the potential to modulate the NP inflammatory microenvironment and restore mechanical stability in IDD. This work represents a straightforward and promising strategy for IDD treatment.

## Introduction

Intervertebral disc degeneration (IDD) is a chronic and multifactorial condition that disturbs the mechanical balance within the IDD [1,2]. This condition is associated with an inflammatory reaction and can lead to long-term persistent low back pain, substantially affecting patients’ quality of life and placing a considerable financial burden on society [3]. The process of IDD is characterized by excessive inflammation and mechanical instability in the nucleus pulposus (NP). The local inflammatory microenvironment of medullary tissues has been extensively investigated, while the study of biomechanical homeostasis has not received sufficient emphasis [4]. The altered biomechanical homeostasis during IDD results in localized stress redistribution, thereby further accelerating the degeneration of the intervertebral disc and adjacent discs [5]. The current clinical treatment protocols fail to adequately address the local inflammation and biomechanical changes associated with disc degeneration, thus falling short of meeting the therapeutic needs of patients [6,7]. Therefore, it is imperative to develop novel therapies aiming to restore the mechanical homeostasis of the IDD.

The NP tissue is enclosed by the annulus fibrosus and adjacent endplates, which possess distinctive viscoelastic properties, enabling the disc to withstand high pressures during daily activities. The high viscoelasticity of NP tissue regulates cellular behavior and contributes to the progression of IDD. The mechanical properties of the NP tissue decrease as the water content decreases during IDD, accompanied by structural damage. The decrease in viscoelasticity is accompanied by a reduction in the levels of collagen type II and glycosaminoglycans within NP. The biomechanical declines not only impact the regular physiological functioning of the body but also expedite IDD. The treatment of IDD offers several surgical procedures; however, none of them effectively restore the normal biomechanics of the motor segments. Although there has been extensive research into NP replacement materials, the development of implants that accurately replicate the mechanical properties of the NP remains a challenge. The utilization of biodegradable materials is a therapeutic approach that heavily relies on the process of cell growth and proliferation to facilitate the repair of degenerated discs. The intricate physiological milieu encountered during routine activities presents a barrier to the extensive application of such materials. The development of a biomaterial that integrates biomechanical restoration and regeneration of the NP tissue is therefore imminent.

Injectable hydrogels are highly promising biomaterials for addressing IDD because of their biocompatibility, structural similarity to biological tissues, and versatile mechanical properties [8,9]. Hydrogels with dual functionalities have been reported previously. They aim to deliver cytokines for local inflammation suppression and serve as scaffolds for NP cell regeneration [10]. The transportation and cell regeneration functions of hydrogels play crucial roles in the treatment of IDD [11]. The self-healing properties of the hydrogel allow it to maintain functional stability in the complex mechanical environment of the disc [12]. The incorporation of additional properties can enhance the suitability of hydrogels for treating IDD. Polyvinyl alcohol (PVA) hydrogel has been widely used to treat skin defects because of its excellent biocompatibility and mechanical properties [13]. With rapid advancements in bioengineering, PVA has gained attention in the field of orthopedics and is considered a promising material for various applications [14]. Wu et al. [15] recently developed a composite PVA hydrogel with exceptional tear strength and toughness, making it suitable for use as an artificial tendon. In addition, PVA hydrogels have been identified as potential substitutes for NP tissues [16]. Therefore, PVA hydrogel shows great potential as a promising biomaterial for treating IDD in the biomedical field.

However, the nondynamic covalent polymer network and long degradation period of pure PVA hydrogels substantially impair their clinical use and curative effects. A strategy was developed to dynamically regulate the stiff network of PVA hydrogels by introducing ions such as K^+^, Na^+^, Cs^+^, Li^+^, Ca^2+^, and Mg^2+^. Among these ions, Mg^2+^ can influence the aggregation states of the polymer chains through the Hofmeister effect, leading to the modulation of the network stiffness [17]. Mg^2+^ plays a crucial role in numerous physiological and biochemical processes in the human body. Previous studies have shown that adequate Mg^2+^ supplementation can effectively suppress local inflammatory responses [18]. Magnesium supplementation is an effective measure of noninfectious inflammatory responses [19]. Therefore, moderate magnesium supplementation of NP tissue during IDD treatment may be a promising therapeutic approach. Thus, developing a magnesium ion-modulated PVA hydrogel material holds great promise for IDD treatment.

**Fig. 1.**
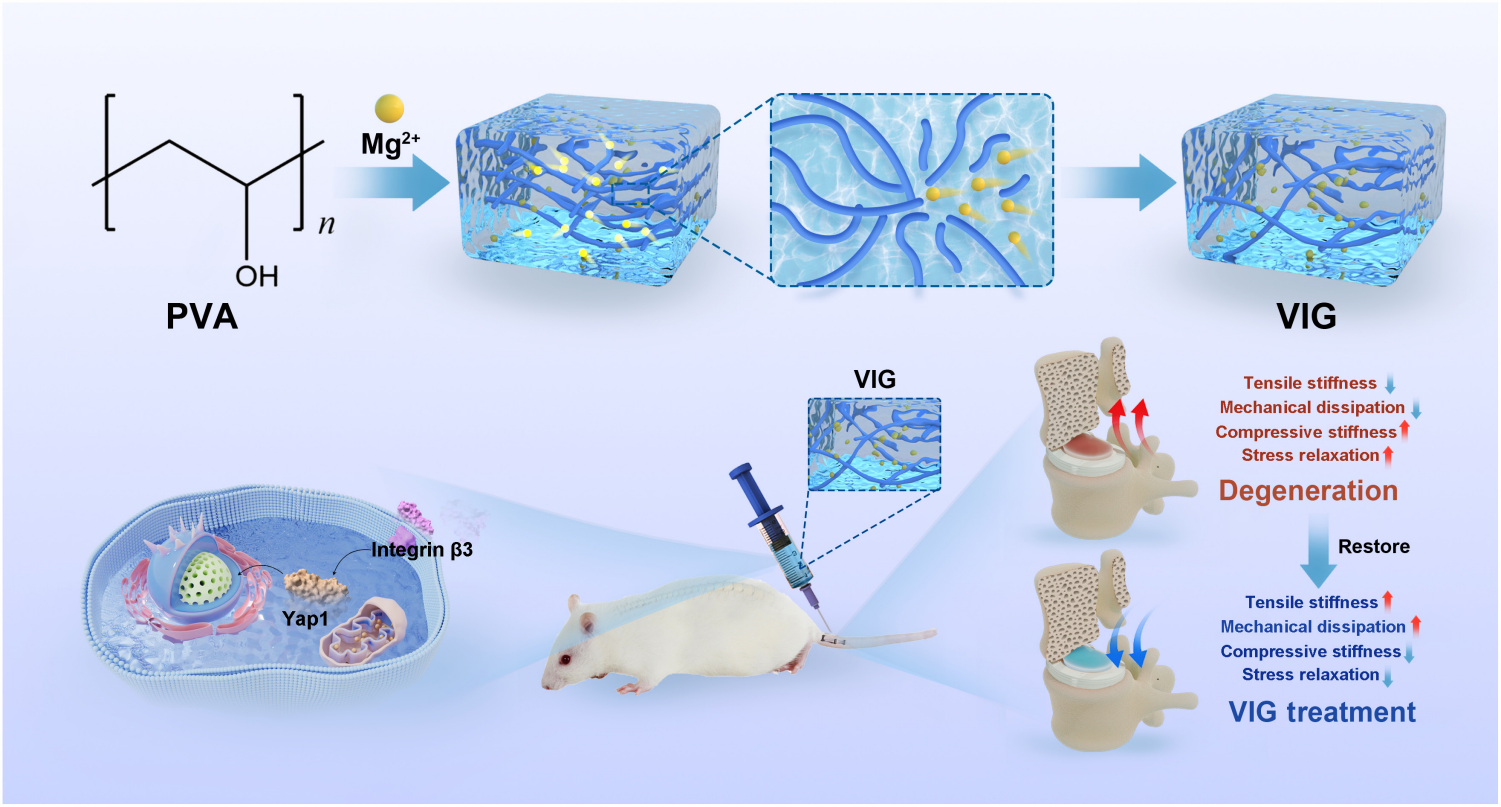
Schematic representation of treating disc degeneration and restoring spinal mechanical homeostasis with injectable VIG.

The present study aimed to develop an injectable viscoelastic ionic hydrogel (VIG) through a physical cross-linking approach, with the objective of restoring mechanical homeostasis in degraded IDD for effective IDD treatment. The physical properties of VIG, such as viscoelasticity, are similar to those of NP tissues, and its ability to attenuate degeneration of NP cells was demonstrated in in vitro and in vivo experimental results (Fig. 1). During an in-depth investigation of the mechanism, it was observed that VIG exhibited a stimulatory effect on extracellular matrix (ECM) synthesis while simultaneously reducing degradation metabolism levels. Furthermore, RNA-sequencing (RNA-seq) results indicated that VIG exerted its action through the YAP-related pathway. VIG has the potential to serve as a reparative material for NP tissues, restoring their mechanical properties, and also as a therapeutic drug to decelerate the degeneration of NP cells. This dual action importantly alleviates IDD and effectively prevents the vicious cycle of degeneration, which reduces mechanical properties. This discovery offers a novel avenue for the treatment of IDD.

## Materials and Methods

### Preparation of hydrogel materials

VIGs with varying stiffness levels were created by adjusting the solid content. These were named low-stiffness VIG (LS-VIG), VIG, and high-stiffness VIG (HS-VIG). First, PVA particles were dissolved in deionized water at 95 °C for 1 h to create polymer solutions with concentrations of 6%, 8%, and 10%. Next, 1.5 wt % magnesium chloride particles were added to the 3 PVA solutions until they dissolved. The resulting mixture was then transferred to a mold and cooled to room temperature. Finally, LS-VIG, VIG, and HS-VIG were created by freezing and thawing 3 types of PVA-Mg^2+^ solutions. The PVA contents of LS-VIG, VIG, and HS-VIG were 6%, 8%, and 10%, respectively. The solutions were cycled thrice, twice, and once. Freezing occurred at −20 °C for 12 h, followed by thawing at room temperature for 2 h. Furthermore, PVA hydrogels were prepared for the control with PVA solid contents of 6%, 8%, and 10 wt % (6% PVA, 8% PVA, and 10% PVA, respectively) using identical freeze–thaw processes for 3, 2, and 1 cycles, respectively.

### Characterization

The microstructure and energy-dispersive spectroscopy (EDS) maps of the freeze-dried samples precoated with the Au–Pa coating were observed using a field-emission scanning electron microscope (SEM; Carl Zeiss, Germany). Infrared spectra were collected using a Nicolet iS20 FT-IR spectrometer (Thermo Fisher Scientific, USA) equipped with a single-bounce diamond stage attenuated total reflectance (ATR) accessory. The resolution was 4 cm^−1^, with a range of 500 to 4,000 cm^−1^, and each sample was scanned 32 times.

### Rheological tests

The rheological properties of the hydrogels were characterized using a Discovery Hybrid Rheometer-2 (DHR) instrument (AR2000, Waters TA Instruments, USA) equipped with a temperature control unit in the oscillatory and flow modes. A plate–plate configuration was used with a 20-mm-diameter plate and a 1-mm gap between the top and bottom plates. Oscillation tests were performed on the hydrogels at 37 °C and 1% strain from 0.1 to 10 Hz to determine the energy storage modulus (*G*′), loss modulus (*G*″), and loss factor (tan δ = *G*″/*G*′). Flow tests were performed to obtain the transient response characteristics of the hydrogel material by subjecting it to shear rates ranging from 0.01 to 100 s^−1^ to determine its steady-state flow profile. Self-healing tests were conducted in oscillatory time mode, cycling between 1% and 1,500% strain for 60 s at 37 °C and 1 Hz, to assess changes in *G*′ and *G*″.

### Mechanical tests

For the compression test experiments, cylindrical samples with an approximate height of 10 mm and diameter of 20 mm were prepared. The samples were positioned on the carrier table of a universal mechanical tester, and the compressive loading unit was lowered until it came into contact with the cylindrical hydrogel. For the cyclic compressive loading–unloading tests, the sample was compressed to a strain of 30% and then unloaded back to 0 at a speed of 10 mm/min. The experiment was conducted 200 times.

VIG was loaded into a 20-ml syringe for the push-injection force test. Once the gel formation was complete, a 25-gauge injection needle was attached to the syringe. The syringe was then securely fixed in a vertical position on the test fixture, aligning both the coronal and sagittal surfaces using a mechanical test inductor. The initial force was set to zero, and the compression mold was lowered until it contacted the syringe pusher. The compression mode was selected as the parameter mode, and the compression speed was set to 10 mm/min to measure the changes in the force values.

### Degradation and dissolution

The mass degradation ratio of VIG was evaluated using the weight method. First, the hydrogel samples were dried to obtain their dry weight (*W*_0_). The desiccated samples were then submerged in deionized water at 37 °C to replicate the physiological setting. At different time points, the samples were removed from the water and redried to determine their dry weight (*W*_1_). Finally, the mass degradation ratio was calculated.

$${\text{DR}}_{m}=\left[\frac{W_{0}-W_{1}}{W_{0}}\right]\times 100\%$$

The mass swelling ratio (SR_m_) of VIG was evaluated using the classical gravimetric method. The hydrogel samples were dehydrated and weighed to determine their initial dry weight (*W*_d_). The dried samples were immersed in distilled water at 37 °C to simulate the physiological environment. At designated time intervals, submerged specimens were extracted and weighed (*W*_s_). The mass swelling ratio was calculated.

$${\text{SR}}_{m}=\left[\frac{W_{s}-W_{d}}{W_{d}}\right]\times 100\%$$

### Measurement of magnesium ion release concentration

To characterize the release behavior of Mg^2+^, we accurately weighed the samples and transferred them into 50-ml centrifuge tubes. They were then immersed in deionized water at 37 °C with a solid-to-liquid ratio of 0.1 g/ml. We collected and replenished the extract solution every day for 7 d. Finally, the Mg^2+^ concentrations were determined using an Agilent 710/715 series inductively coupled plasma atomic emission spectrometer (Agilent 710 ICP-OES, Agilent Technologies, USA).

### Animal surgery

Animal procedures were approved by the Ethics Committee of Soochow University (approval no. SUDA20230802A01). Before the surgical procedure, the animals underwent a 12-h fasting period and a 4-h dehydration period. Male Sprague–Dawley rats weighing 300 g were anesthetized using a 2% (w/v) pentobarbital solution at a dose of 100 mg/kg. A 21-gauge needle tip was used to puncture the sixth caudal IDD, with the depth of the puncture being three-quarters of the tail diameter. The needle was rotated for 12 s and then immobilized for 18 s. Three days later, VIGs were injected through the same injection sites and the NP tissue content for VIG injection was calculated separately [20].

### Biomechanical test methods and parameters

After separating the intervening IDD from the upper and lower vertebral bodies, we measured their long and short diameters. The upper and lower vertebrae were then fixed to a universal mechanical tester (HY-0580, Shanghai Heng Yi Co. Ltd., China) to ensure that they remained in the same straight line as the crown-sagittal plane of the test fixture. The tensile–compression cycle test parameters were set to a tension of 0.5 N, compression of 1.5 N, and frequency of 0.5 Hz. Twenty-four cycles were performed to measure the changes in the force values during the stress relaxation test. The test parameters included compression at 1.5 N, holding for 600 s, and measuring the change in the force value.

### Cell extraction and culture

Six-week-old male Sprague–Dawley rats were euthanized by an intraperitoneal injection of 1% pentobarbital. The skin was disinfected with a 10-min soak in 75% alcohol. Under sterile conditions, the IDD tissue was exposed by excising the tail from the fourth caudal vertebra and removing skin and soft tissue. The NP tissue was collected from the IDD and placed in F12 medium supplemented with 4% penicillin–streptomycin. After centrifugation at 1,200 rpm, the supernatant was discarded. Next, 2% type II collagenase was added for digestion, and the mixture was incubated at 37 °C for 3 h. The resulting suspension was passed through a 70-μm cell sieve to remove any remaining tissue. The NP cells were then placed in petri dishes and incubated in a controlled environment at 37 °C in an atmosphere containing 5% CO_2_. Primary cell culture was established by refreshing the culture medium every 48 h until the cells reached approximately 90% confluence in the dish. Once the cells reached this stage, they were briefly treated with trypsin-EDTA solution (5%) for 1 min to detach them from the dish surface. The resulting single-cell suspension was evenly distributed into 2 new culture dishes to establish a passage one-cell generation. The cells were passaged twice before use in subsequent experiments.

### Cell proliferation, viability, and apoptosis staining

The nucleus pulposus cells (NPCs) were cultured in 96-well plates at a seeding density of 2 × 10^3^ cells per well. In the cell experiments, sterilized VIG was injected directly into the wells containing NP cells and a sterilized glass disc slightly smaller than the diameter of the well was placed in each well. Following incubation with various treatments for 24 and 72 h, cell viability was evaluated using Cell Counting Kit-8 (CCK-8). In the CCK-8 experiments, the control group received only the sterilized glass discs, while the LS-VIG, VIG, and HS-VIG groups were all cultured with the respective materials in the manner described. Each well was supplemented with 90 μl of serum-free complete medium and 10 μl of CCK-8 reagent solution. The plate was then incubated at a constant temperature and humidity of 37 °C for 2 h. Cell viability was assessed by measuring the absorbance at 450 nm using the iMark Microplate Absorbance Reader (Bio-Rad, 1681130) and was compared to that of the control group.

To conduct a viability assay on cover glasses, NP cells were treated with IL-1β and VIG separately for 24 h. The Calcein/PI Cell Activity and Cytotoxicity Assay Kit was used to stain the cells. Live cells were labeled green with calcein AM, which is membrane-permeable, whereas dead cells with disrupted membrane integrity were labeled red with propidium iodide (PI), a red fluorescent dye that cannot penetrate intact membranes. Fluorescence staining images were visualized and captured using a fluorescence microscope (Axio Observer, Zeiss, Germany).

### Immunofluorescence and image analysis

NP cells were cultured on cover glasses. The different groups of cells were treated with IL-1β and VIG for 24 h. The cells were washed once with phosphate-buffered saline (PBS) and fixed for 10 min with 4% paraformaldehyde. This was followed by treatment with QuickBlock Immunostaining Confinement Solution adding 0.3% Triton X-100 for 1 h at room temperature. After washing 3 times with PBS, primary antibodies [collagen II, Acan, matrix metalloproteinase-13 (MMP-13), Yap1, Ctnnb1] were added and incubated overnight at 4 °C. After 3 washes with PBS, secondary antibody was added and incubated for 1 h. Finally, the plates were blocked using an antifade mounting medium containing 4′,6-diamidino-2-phenylindole (DAPI). The fluorescence staining results were observed and imaged using a fluorescence microscope.

### Western blotting

A density of 2 × 10^5^ NP cells per well was used to inoculate 6-well plates. After the cells reached 90% confluence, they were homogenized in radioimmunoprecipitation assay (RIPA) lysis buffer and then centrifuged to remove cell debris. Twenty micrograms of total protein was extracted from each experimental group, analyzed by SDS-polyacrylamide gel electrophoresis (PAGE), and then transferred to a nitrocellulose membrane using electrotransfer. The membranes were then washed thrice with 20 mM tris-buffered salt solution containing 1‰ Tween 20 and subsequently incubated with 5% blotting grade for 1 h at room temperature to block nonspecific protein binding. Horizontal strips were cut from the nitrocellulose membranes using molecular weight markers. Each strip was incubated overnight at 4 °C with the corresponding primary antibody. After washing thrice with TBST (tris-buffered saline with Tween 20), the nitrocellulose membranes were incubated for an additional hour with secondary antibodies. Protein fluorescence was induced using an Enhanced ECL Chemiluminescent Substrate Kit, and luminescence was observed using a ChemiDoc Imager (Bio-Rad, USA). For proteins with similar molecular weights, the nitrocellulose membrane was chemically exposed, as described above. After exposure, the membrane was incubated with NCM Western Blot Stripping Buffer for 15 min. The process of blocking, incubation with antibodies against other similar molecular weight proteins, and subsequent incubation with secondary antibodies were then repeated, as mentioned earlier. Protein expression levels were analyzed using ImageJ software developed by the National Institutes of Health (Bethesda, MD, USA) and normalized to β-actin.

### PCR

Cells were seeded in 6-well plates at a density of 5 × 10^5^ NP cells per well. Once the cells reached 90% confluence, total RNA was extracted using TRIzol reagent. The resulting extract was treated with chloroform, followed by RNA precipitation with isopropanol. After washing the total RNA precipitate 3 times with 75% ethanol and allowing to dry, it was dissolved in diethyl pyrocarbonate (DEPC) water. Following the instructions provided with the reverse transcription kit, RNA, MIX, and DEPC water were combined in EP (Eppendorf) tubes for reverse transcription using a dedicated instrument. The polymerase chain reaction (PCR) amplification process involved the addition of cDNA, MIX, and primers according to the specified guidelines. Subsequently, quantitative real-time fluorescence PCR was performed using a real-time PCR amplifier (Bio-Rad, Hercules, CA, USA) under predefined conditions.

### Histological staining

Rat spine samples were fixed in 4% paraformaldehyde solution for 48 h. Subsequently, decalcification was performed using a 15% ethylenediaminetetraacetic acid (EDTA; pH 7.4) solution for approximately 4 weeks. The decalcified samples were embedded in paraffin, and mid-sagittal sections of 4-μm thickness were prepared for histological staining. Morphological changes in the tissues were evaluated using routine hematoxylin and eosin (H&E) staining. Collagen distribution within NP tissue was assessed using Safranin O and Fast Green staining. Immunohistochemical analysis was conducted using antibodies against collagen II, Acan, and MMP-13 to evaluate protein expression and distribution in the NP.

### Transcriptome sequencing

NP cells induced by interleukin-1β (IL-1β) were treated with VIG. After a 24-h incubation period, cells from different experimental groups were individually harvested, and total RNA was extracted using the TRIzol reagent. The RNA samples were quantified using a NanoDrop 2000 spectrophotometer (Thermo Fisher Scientific, USA). Transcriptome sequencing was conducted by the Shanghai OE Biotech Co. The clean read lengths were aligned to the reference genes using the Hisat2 software. Fragment values per kilobase per million mapped reads (FPKM) were calculated using the Cufflinks software. Differentially expressed transcripts were evaluated for marked differences using Gene Ontology (GO) and the Kyoto Encyclopedia of Genes and Genomes (KEGG) enrichment analyses. Genes with *P* values below 0.05 and fold changes exceeding 1.5 were deemed statistically significant in relation to their expression levels. Unsupervised hierarchical clustering was used to analyze the differential transcripts presented in a heatmap format.

### Finite element analysis

Spinal DICOM and Communications in Medicine data were acquired from young individuals with no history of lumbar disc herniation. The data were imported into the MIMICS software version 21.0, where the bone, disc, and NP were segmented by defining a range of pixel values. A region-growing algorithm was used to purify pixels of each tissue. The calculated 3-dimensional (3D) commands are executed to generate a smooth 3D contour. The binary STL file was exported and converted into a 3D solid geometry model using Geomagic Studio. This model was then imported into the COMSOL Multiphysics software to establish material models and parameters, load boundary conditions, and mesh dissections for mechanical simulation calculations. The calculation results are presented in Table.

**Table.** Material properties and element types (name in Abaqus software) used for various structures in the normal model

Tissue	Young’s modulus (MPa)	Poisson’s ratio	Element type	Number of element	Ref.
Bone	12,000	0.3	Linear-elastic tetrahedron	41,326	[42]
Annulus fibrosus	295	0.35	Linear-elastic tetrahedron	3,358	[43]
Nucleus pulposus	1	0.499	Viscoelastic tetrahedron	226	[44]

### Data analysis

The data were validated using multiple independent sample experiments (*n* ≥ 3). Two independent sample *t* tests were conducted using SPSS for statistical analysis. Graphical data were plotted using Origin and GraphPad Prism. In the analysis of variance, * denotes a significance level of *P <* 0.05, ** represents *P <* 0.01, and *** indicates *P <* 0.001.

## Results and Discussion

### Characterization and physical properties of VIGs

#### Microstructure of VIG

**Fig. 2.**
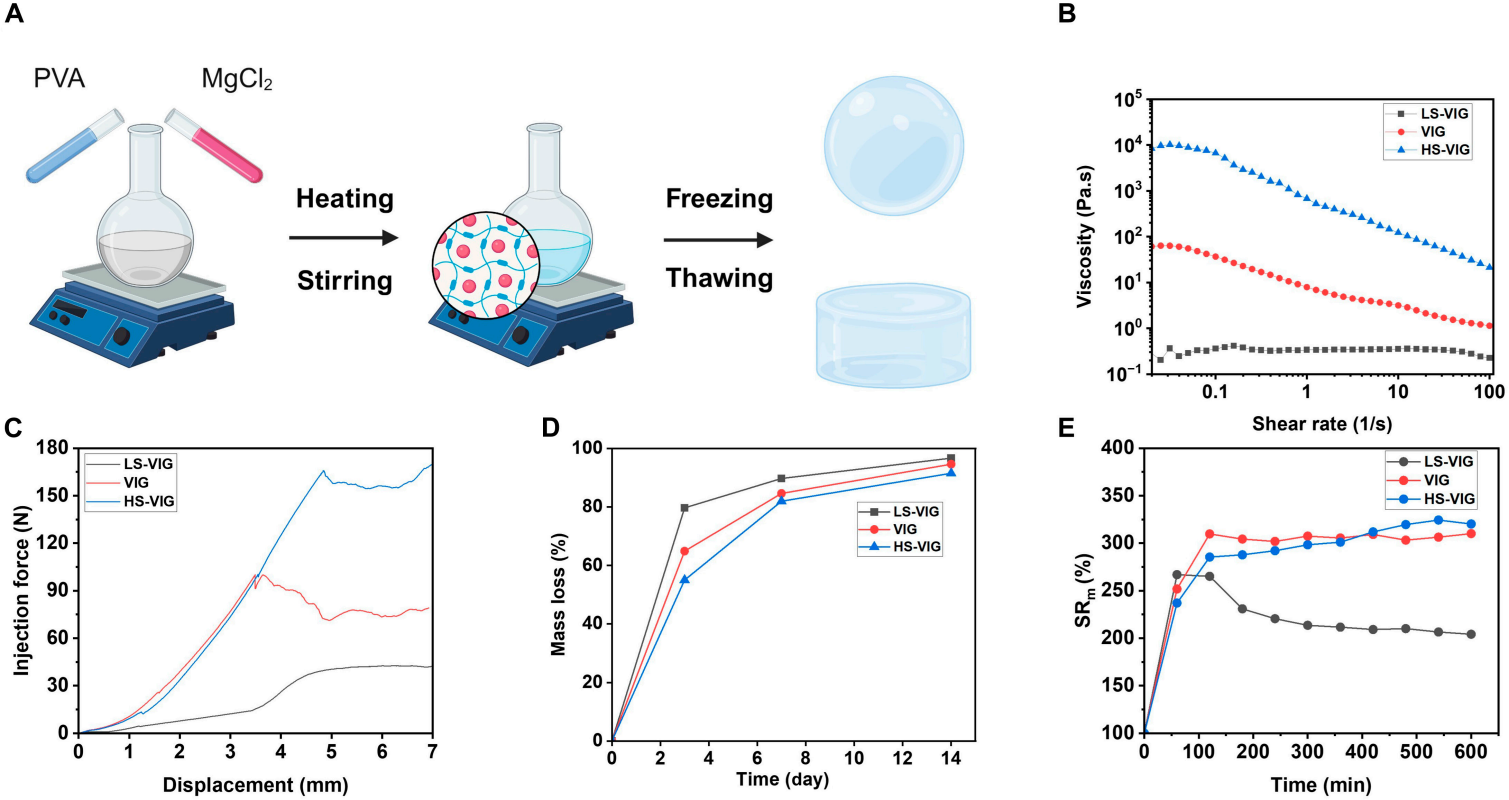
Preparation and characterization of the injectable VIG. (A) Schematic diagram of the preparation of VIG. (B) Degradation of VIG within 2 weeks. (C) Dependence of the viscosity of VIG in a shear rate range of 0.1 to 100 at 37 °C. (D) Injection force of VIGs during passing 25-gauge needles. (E) Swelling ratio in mass of VIGs.

Viscoelastic hydrogels that could be injected were prepared using a one-pot method. The synthetic route to the viscoelastic hydrogel is shown in Fig. 2A. Fourier transform infrared (FTIR) spectroscopy (Fig. S1) showed the characteristic stretching bands of O–H at 3,263.2 to 3,299 cm^−1^ in pure PVA hydrogels with solid contents of 6, 8, and 10 wt %. Upon adding Mg^2+^, VIGs exhibited little change in the stretching bands of O–H, ranging from 3,206.4 to 3,220.6 cm^−1^. This indicates that Mg^2+^ only modifies the physical crosslinking of PVA hydrogels without altering their chemical structure. Unlike most studies that enhance hydrogel properties by chemically modifying covalent bonds, this approach avoids the cytotoxic reactions often associated with changes to chemical bonds [21–23]. Moreover, by exclusively altering physical crosslinking, the biocompatibility of PVA hydrogels is minimally affected.

#### Rheological properties of VIG

**Fig. 3.**
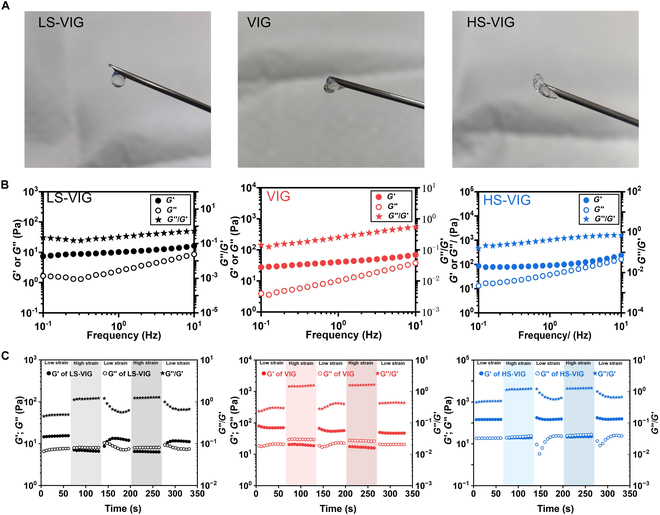
Characterization of the injectable VIG. (A) Photograph of the injected VIGs. (B) Dependence of *G*′, *G*″, and the *G*″/*G*′ ratio (loss factor) of VIGs on the frequency of oscillation. (C) Self-healing property of VIGs characterized by rheometer at the alternate step strain switched from 1% to 1,500%, frequency of 1 Hz, and at 37 °C.

Rheological tests were conducted in the oscillation mode to study the viscoelastic properties of the hydrogels. As shown in Fig. 3B, all 3 VIGs exhibited higher *G*′ values than *G*″, and their *G*′ increased from 7.42, 27.46, and 77.22 Pa to 16.32, 69.70, and 237.39 Pa with increasing frequency, indicating their behavior as viscoelastic solids with a characteristic frequency-dependent stiffness, which was similar to the shock-absorbing properties of natural NP tissue. Furthermore, it was worth mentioning that *G*″ of all the VIGs exhibited a stronger frequency-dependent property, with a noticeable increase of *G*″ from 1.66, 3.93, and 13.13 Pa to 8.64, 37.66, and 163.40 Pa as the frequency increased from 1 to 5 Hz, corresponding to normal physical activities ranging from normal walking to running.

#### Injectability of VIG

The shear viscosity analysis of 3 VIGs showed a gradual decrease in viscosity from 0.32, 36.62, and 6,661.40 Pa·s to 0.23, 1.13, and 21.28 Pa·s with increasing shear rate from 0.1 to 100 s^−1^ (Fig. 2B). The VIGs demonstrated characteristic shear-thinning properties during injection (Fig. 3A). The study found that the 3 VIGs had injection forces of 41, 75, and 157 N during their passage through a 25-gauge needle (Fig. 2C).

#### Self-healing and stability of VIG

The rheological properties of the hydrogels were tested for viscoelastic and structural recovery after a high shear strain was applied. Figure 3C shows that the VIGs exhibited that *G*″ values exceed *G*′ under high shear strain. Upon returning to the original low strain, the *G*″ of the VIGs instantaneously returned to their initial values, whereas the *G*′ of the VIGs was restored to 83.5%, 89.6%, and 86% of their initial values. After injection from the syringe, the VIGs could withstand cyclic loadings similar to those of the pure PVA hydrogel, and the stress–strain correlation remained nearly unchanged after hundreds of loading/unloading cycles (Fig. S2).

#### Characterization of localized release of Mg^2+^ by VIG

**Fig. 4.**
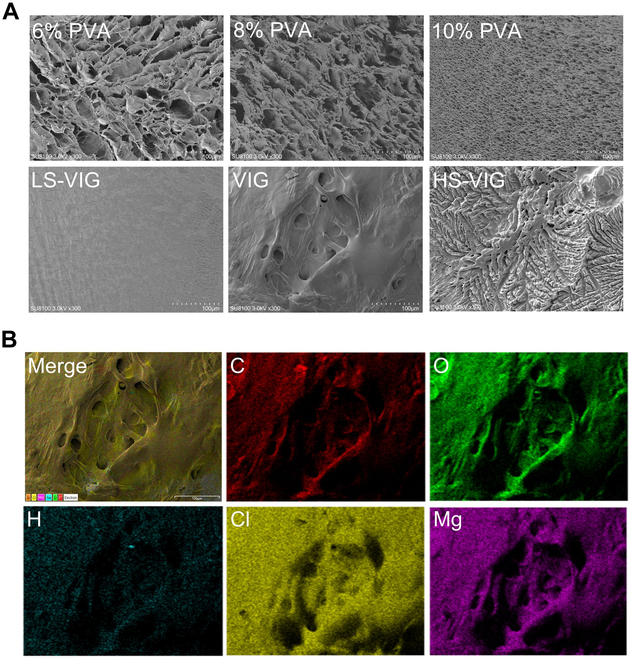
Microstructure of the injectable VIG. (A) SEM images of freeze-dried hydrogels. (B) SEM mapping images of VIG. Scale bar, 100 μm.

The mass loss ratios of LS-VIG, VIG, and HS-VIG were 96%, 94%, and 91%, respectively, on day 14 of immersion in deionized water, based on the degradable properties of the hydrogels (Fig. 2D). In contrast, pure PVA-based hydrogels exhibited slow degradation rates both in vivo and in vitro. The swelling behavior of the hydrogels was investigated. Based on the swelling test results, VIG and HS-VIG had a higher rate of water absorption than LS-VIG (Fig. 2E). To further understand the microarchitecture of the hydrogels, SEM was used to study the morphology of the polymer network by observing the cross-sections of the hydrogels (Fig. 4A). The PVA hydrogel had a clear network structure composed of polymer chains, whereas LS-VIG had a dense bulk structure with few pores formed by frozen water during the drying process. Furthermore, the EDS mapping image of VIG displayed a uniform distribution of hydrated Mg^2+^ (Fig. 4B), indicating its strong hydration properties. This study evaluated the release kinetics of Mg^2+^ from VIGs by immersing the samples in deionized water for a maximum of 7 d to assess their impact on biological activities. The hydrogels exhibited effective Mg^2+^ release on the first day with minimal degradation (Fig. S3). This releasing behavior enabled VIGs to maintain a stable concentration of Mg^2+^ within the IDD for a specific duration after injection.

#### Biocompatibility of VIG

The biocompatibility of VIGs was tested. The extracts were cocultured with the cells for 24 h, and no substantial effects on cell proliferation were observed (Fig. S4A). When cells were cocultured directly over VIG, a major proportion of live cells and only a small number of dead cells were observed in the control, LS-VIG, and VIG groups. However, a higher percentage of cell death was observed in the HS-VIG group (Fig. S4B and C). These results suggested that the Ctrl, LS-VIG, and VIG groups had minimal impact on cell activity. Furthermore, pathological analysis of various rat tissues indicated that VIG caused no marked harm to rats (Fig. S4D).

The human body contains many viscoelastic tissues, including NP [24]. Previous studies have shown that biomaterials with viscoelasticity can provide stronger guidance for cell behavior [24]. Indana et al. [25] found that the proliferative viabilities and differentiation directions of human-induced pluripotent stem cells were influenced by modulating the viscoelasticity of hydrogel materials. This study indicates that rapid stress relaxation has a positive effect on cells, and that the YAP protein plays a role in viscoelastic sensation. Researchers have extensively studied the use of viscoelastic materials for tissue repair in various parts of the human body [26]. The rheological similarity of VIG to NP tissue, its ease of injection via syringe, stability of physical properties after repeated compression, local sustained release of metal ions, and excellent biocompatibility make it highly advantageous for clinical applications.

### Finite element analysis of VIG optimizing the pathological stress environment of degenerated IDD

**Fig. 5.**
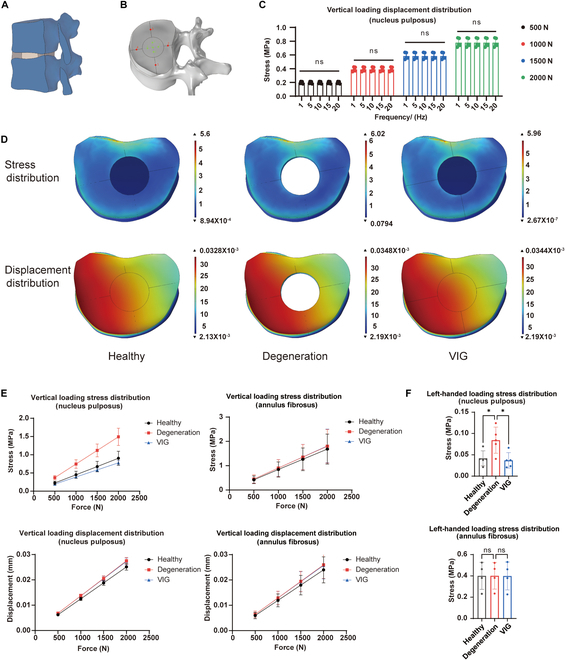
Simulation of finite element analysis of VIG for in vivo application in humans. (A) Construction of a finite element model of the human lumbar spine. (B) Test point locations located in the NP and annulus fibrosus mechanics during finite element simulation of intervertebral disc stress. (C) Average stress distribution on the NP at different frequencies of vertical loading. (D) Distribution of intervertebral disc stress and displacements in healthy, degenerative, and VIG treatment groups under vertical loading. (E) Quantitative analysis of the stress and displacement distribution in NP and annulus fibrosus under vertical loading. (F) Quantitative analysis of the stress distribution in NP and annulus fibrosus under left-handed loading. *P < 0.05, **P < 0.01, and ***P < 0.001.

A tetrahedral model was constructed to conform to the finite element model of the human spine, based on the data in the previous study (Fig. 5A and Table). Simultaneously, we selected 5 points in the NP and 4 points in the annulus fibrosus (Fig. 5B) to describe the average force and displacement of the structures. The frequency experiments revealed that the discs in the VIG group did not demonstrate significant alterations in kinematic characteristics when subjected to mechanical loading ranging from 1 to 20 Hz (Fig. 5C and Fig. S5A). Therefore, we compared the data from the 10-Hz mechanical loading with the healthy and degeneration groups in the subsequent analysis.

After applying vertical stresses ranging from 500 to 2,000 N, the force at the NP site was significantly higher in the degeneration group, indicating a decrease in mechanical cushioning of the disc. The force distribution in the VIG group was superior to that in the degenerative disc group, suggesting that VIG effectively restored the mechanical cushioning capacity of the disc (Fig. 5D). The force measurements of NP component did not show any statistically significant differences between the VIG and healthy groups at the same time. The implication here is that the VIG has the potential to serve as a substitute for the mechanical functions of the NP within the intervertebral disc. Displacement measurements at the NP locus showed no significant changes. No significant differences were found in the stress and displacement measurements at the annulus fibrosus locations among the 3 groups (Fig. 5E and F). Similar results were observed for the horizontal stress and rotation (Fig. S5B to D). Regarding stress distribution, the degeneration group exhibited significantly higher mechanics at the NP site, which closely resembled the mechanical distribution of the healthy group, where VIG repaired the absence of NP in the mechanical structure of the disc.

This approach addresses the limitations of previous studies, which were predominantly conducted either in vitro or in animal models [27,28]. The loss factor of the VIG increases proportionally with frequency, which enhances its pressure cushioning effect in vivo as exercise frequency increases. Finite element analysis further demonstrated the protective effect of VIG on the overall mechanical distribution of the intervertebral disc.

### Effect of VIGs on restoring biomechanical properties of degenerated IDD

**Fig. 6.**
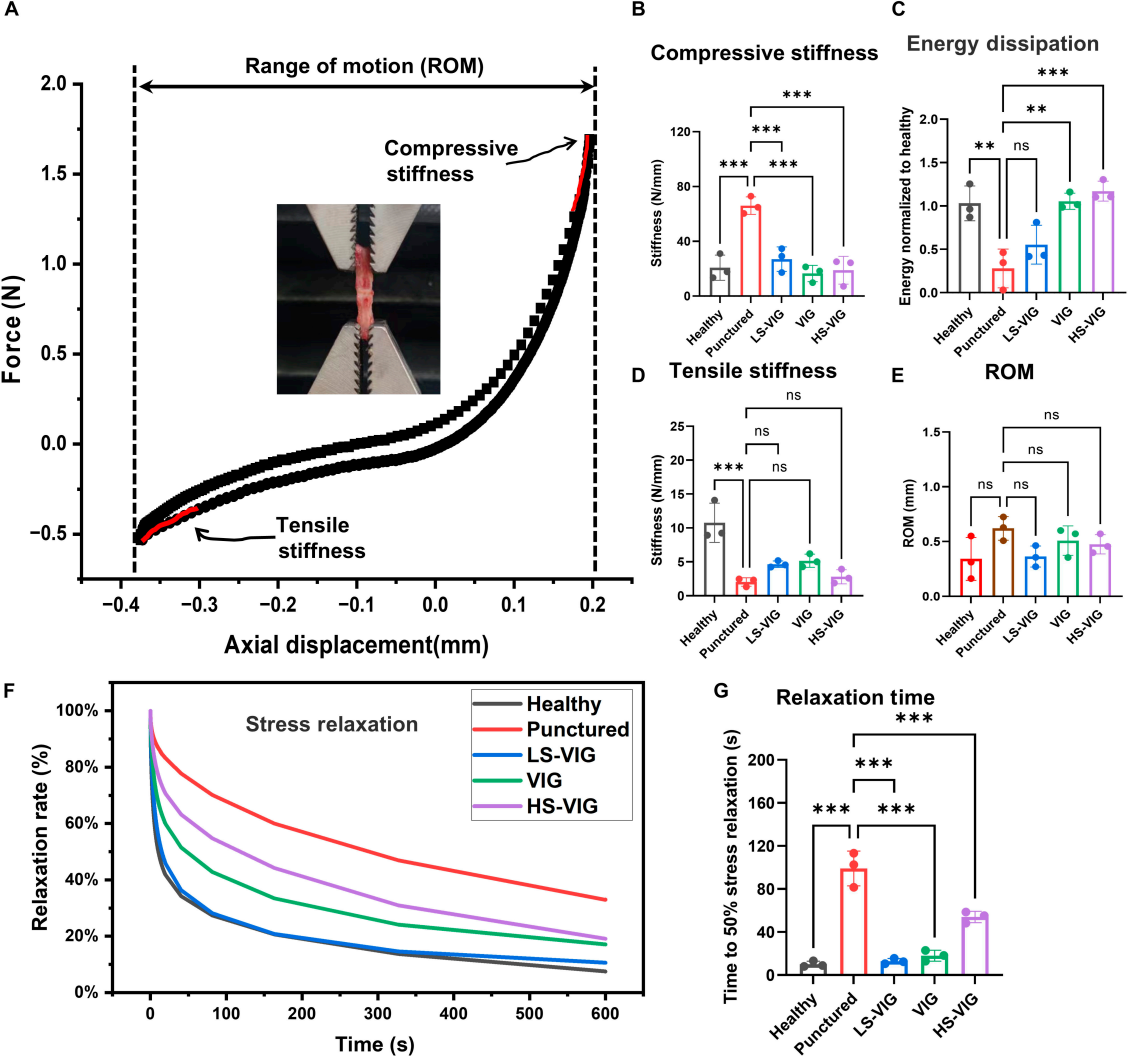
Mechanical performances of rat caudal tissue. (A) Schematic representation of mechanical testing of rat caudal tissue. Compressive stiffness (B), energy dissipation (C), tensile stiffness (D), and range of motion (ROM) (E) of the rat caudal in different treatment groups. (F) Stress relaxation curves in the rat caudal spine. (G) Time taken for stress relaxation to decrease by half in the caudal spine of rats. *P < 0.05, **P < 0.01, and ***P < 0.001.

The mechanical properties of rat caudal IDDs were measured using the test setup shown in Fig. 6A. After 50 compression–tension cycles, the punctured group exhibited a decrease of approximately 70% in tensile stiffness compared with the healthy disc, whereas a significant increase in compression stiffness was observed (Fig. 6B and D). All 3 concentrations of VIG in the material treatment group improved the tensile and compressive properties of intervertebral discs. However, there was no statistically significant difference in the range of motion of the intervertebral discs under isometric force. The absolute value of the range of motion of the intervertebral discs in the material group showed some improvement compared to that in the degeneration group (Fig. 6E). After calculating the energy density per unit volume, LS-VIG and VIG improved the energy density of the degenerated discs and restored their energy-cushioning functions (Fig. 6C). The stress relaxation curves with 1/2 stress relaxation time also showed a significant decrease in the viscoelasticity of the discs in the degeneration group, and the VIG treatment was more effective in restoring the viscoelasticity of the degenerated discs (Fig. 6F and G).

The test results aligned with the mechanical changes observed in the intervertebral disc during the progression of IDD [29]. This process involves a decrease in disc height, an increase in intravertebral disc pressure, a shift in maximum compressive stress, and an increase in stiffness under load. As the IDD loses mechanical stability, osteophyte formation, endplate chondrosclerosis, and disc herniation develop [30,31]. These changes disrupt the mechanical equilibrium of the disc, resulting in reduced range of motion during extension, flexion, and rotation [32]. This perpetuates disc degeneration, creating a detrimental cycle [33]. When disc degeneration worsens, the mechanical stability of the spine is compromised [34]. Chronic loading caused by the IDD can have severe effects on the spine [35]. Therefore, it is important to focus on restoring mechanical stability during IDD to promote recovery of spinal function and restore spinal stability in patients with IDD. The hydrogel described in this study can be injected into degenerated NP tissue. Through a similar swelling effect, it can replace NP with their responsive mechanical function. This facilitated the overall mechanical restoration of the degenerated disc, as demonstrated in the in vivo experiments. Restoration of mechanical homeostasis effectively terminates the vicious cycle caused by degeneration and delays IDD.

### Effects of VIGs on inhibiting IDD progression in vivo

**Fig. 7.**
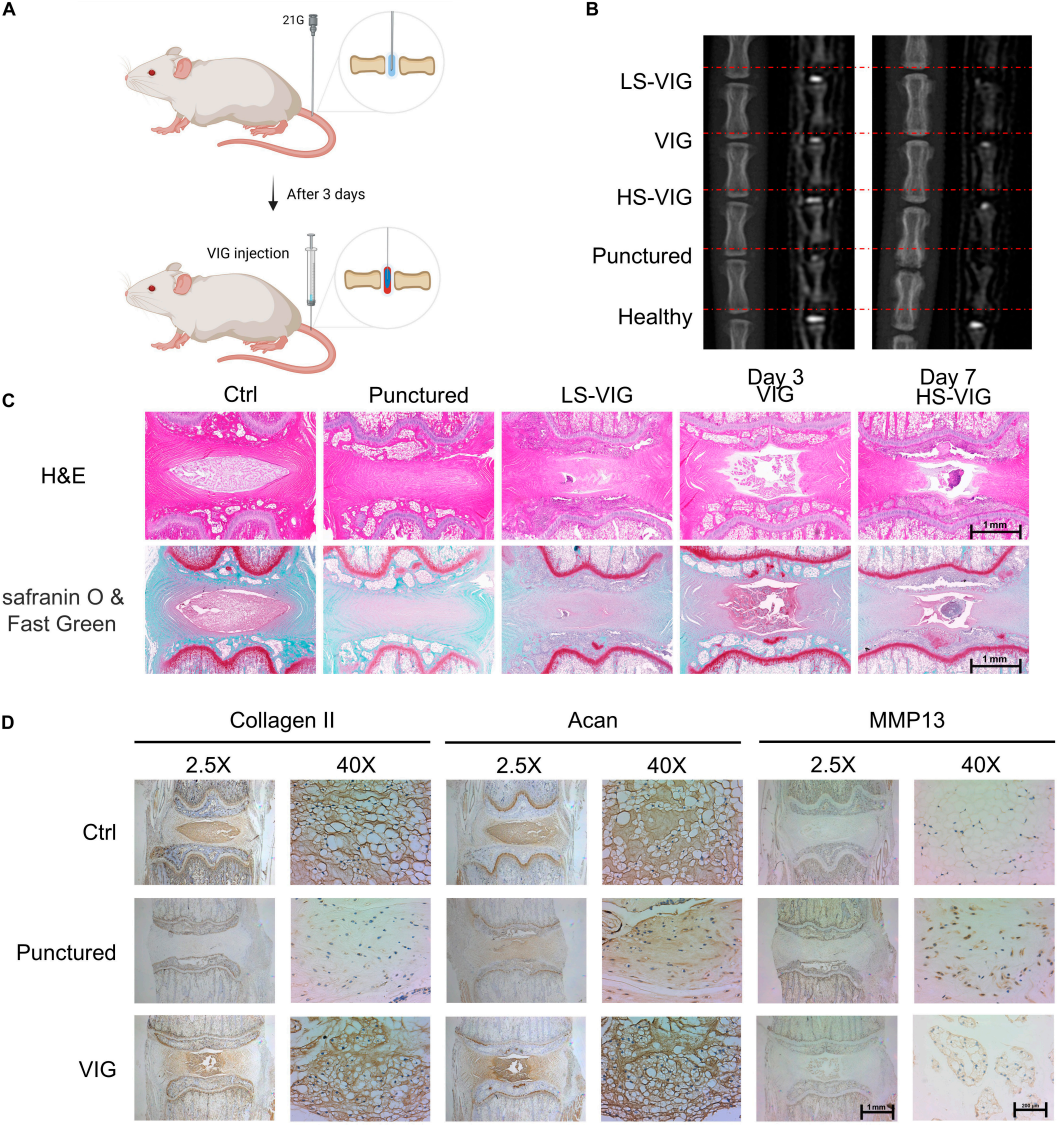
Radiological evaluation and histological analysis of animal experiments. (A) Schematic illustration of the construction of a rat caudal disc needling model and minimally invasive treatment using VIGs. (B) X-ray images and MRI of IDDs at 3 and 7 d after surgery. (C) H&E and Safranin O/Fast Green staining images of IDDs at 7 d postoperatively. Scale bar, 1mm. (D) Immunohistochemical staining analysis of collagen II, Acan, and MMP-13 in IDD tissues. Scale bar, 200 μm.

In the in vivo experiments using a rat model (Fig. 7A), magnetic resonance imaging (MRI) of the rat caudal tissues indicated that the water content of the NP in the puncture group decreased by the third day after treatment, whereas the NP in the VIG group maintained better water content. On day 7 after treatment, the NP tissue in the punctured group and LS-VIG groups appeared almost dehydrated, with T2 hypointense signal. The NP in the VIG group and HS-VIG group still had T2 hyperintensity, indicating that some of the NP tissues contained water (Fig. 7B). The discs in the VIG group had a lower grade 2 Pfirrmann score on days 3 and 7 than other groups. On day 7, the disc height in the VIG group showed slower collapse than that in the puncture group (Fig. S6A).

Our MRI observations were confirmed by H&E and Safranin O/Fast Green staining, which showed that NP degeneration was milder in the VIG and HS-VIG groups than in the punctured group (Fig. 7C). The NP in the VIG group was more intact, and a collagen-rich matrix was observed. In both the LS-VIG and punctured groups, the NP was degenerated, and the tissue sections showed fibrosis of the NP tissue, making the typical NP tissue structure unobservable. Immunohistochemical staining (Fig. 7D) revealed an approximately 50% increase in the expression of collagen II and Acan in the VIG group compared with that in the punctured group. Furthermore, the expression of MMP-13 in the VIG group was half of that in the punctured group (Fig. S6B).

In vivo animal experiments confirmed the alleviating effect of VIG on IDD. This effect is associated with the early and sustained mechanical properties provided by VIG and the localized, prolonged release of magnesium ions. The role of magnesium ions in IDD has been previously studied. In the work by Jiang et al. [36], a hydrogel material that locally releases Mg^2+^ was designed to alter the expression of local macrophages in degenerated intervertebral discs, thereby alleviating IDD. These studies suggest that Mg^2+^ holds significant potential for the treatment of IDD. In this study, VIG released Mg^2+^ during degradation, and these Mg^2+^ ions, which are crucial components of human metabolism, may interact with the NP tissues. Zhang et al. [37] showed that Mg has significant antioxidant effects and can mitigate the degeneration and apoptosis of NP cells. The presence of Mg^2+^ effectively reduces the levels of inflammatory factors, thereby improving the inflammatory microenvironment in IDD. Simultaneously, the hydrogel carrier ensures sustained release and local maintenance of the Mg^2+^ concentration [38]. The VIG hydrogel system loading and sustained release capabilities greatly facilitate IDD treatment. This suggests that VIG can serve a similar function in managing IDD.

### Effect and mechanism of VIGs on regulating anabolism and catabolism of NPCs

**Fig. 8.**
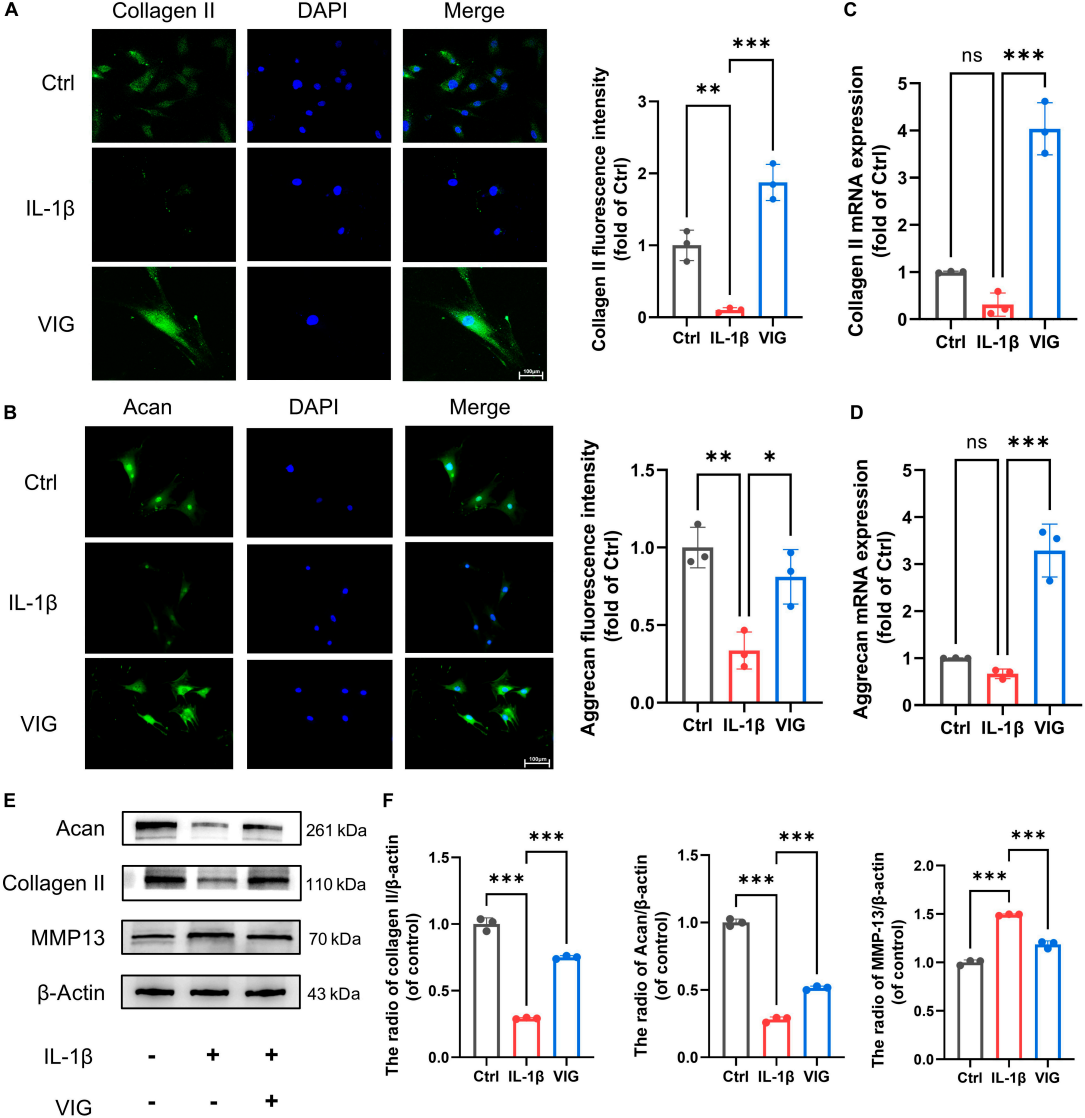
Characterization of VIG intervention in ECM metabolism. Fluorescent staining and semiquantitative analysis of collagen II (A) and Acan (B) proteins in NP cells. RT-PCR results of relative mRNA levels of collagen II (C) and Acan (D) in NP cells after treatment with VIG. (E) Collagen II, Acan, and MMP-13 proteins were measured by Western blotting in NP cells treated with VIG for 24 h. (F) Quantitative analysis of collagen II, Acan, and MMP-13 proteins detected by Western blotting. Scale bar, 100 μm. *P < 0.05, **P < 0.01, and ***P < 0.001.

IL-1β was used to simulate NP degeneration in cells. In the cell fluorescence staining, the fluorescence intensity of collagen II and Acan in the IL-1β group was approximately 30% of that in the control group. The intracellular expression levels of collagen II and Acan in the VIG group were similar to those in the control group (Fig. 8A to D). MMP-13 was highly expressed in the IL-1β group, indicating ECM degradation (Fig. S7A and B). The findings of this study provide evidence that VIG effectively attenuates the IL-1β-induced degradation of the ECM (Fig. 8E and F).

**Fig. 9.**
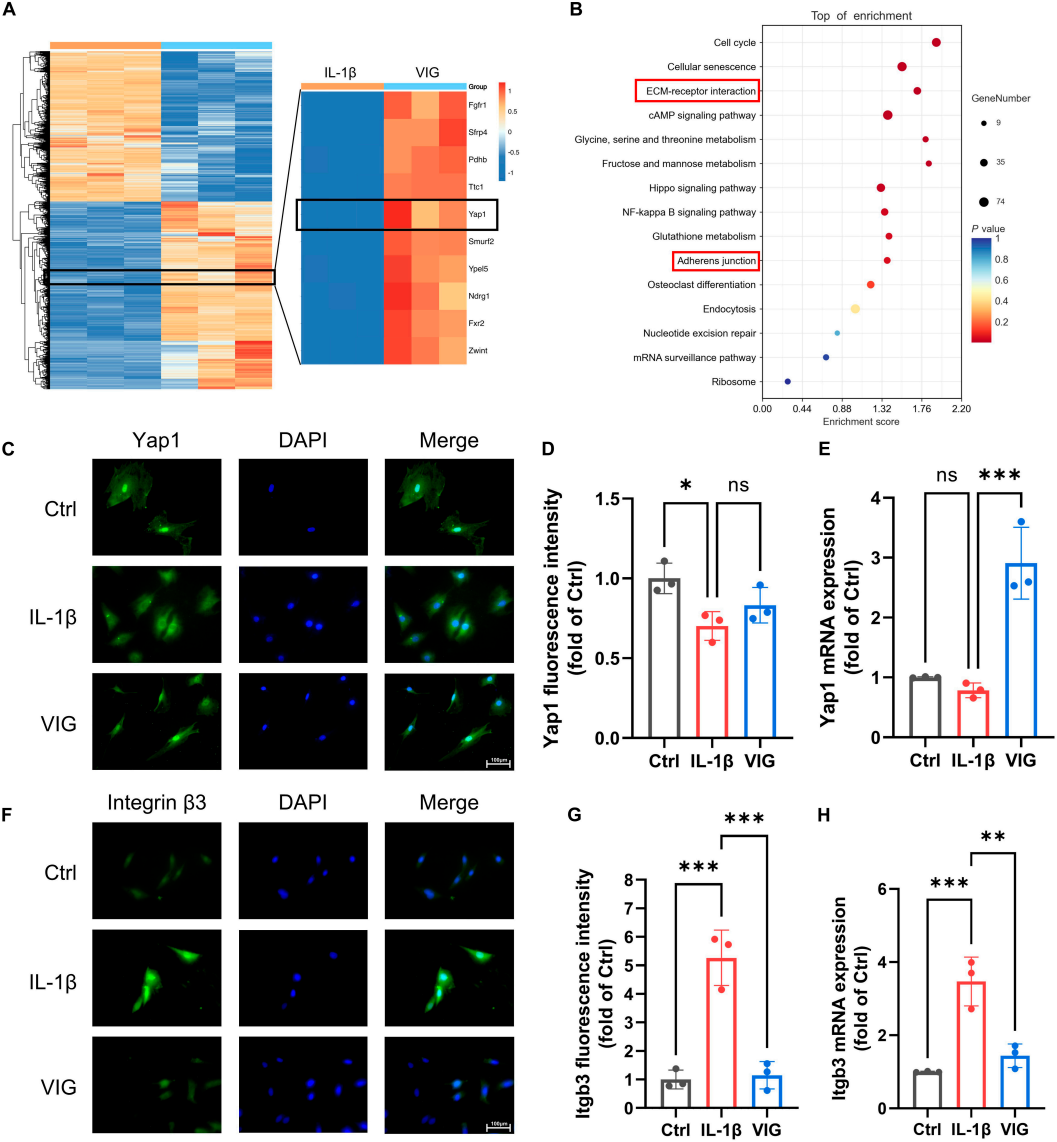
Transcriptome sequencing analysis of VIG group and Ctrl group cells. (A) Heatmap of differentially expressed genes. (B) KEGG enrichment analysis of differentially expressed genes. Fluorescent staining of Yap1 (C) and Itgb3 (F) proteins in NP cells. Semiquantitative analysis of Yap1 (D) and Itgb3 (G) proteins in NP cells. RT-PCR results of relative mRNA levels of Yap1 (E) and Itgb3 (H) in NP cells after treatment with VIG. Scale bar, 100 μm. *P < 0.05, **P < 0.01, and ***P < 0.001.

RNA-seq was performed on cells from the IL-1β and VIG groups to investigate alterations in the transcriptome expression in NP cells. The aim was to explore the molecular mechanisms that regulate the function of NP cells to antagonize IL-1β under hydrogel-loading conditions. Using the IL-1β group as a control, we detected 2,230 overexpressed genes and 1,459 genes with significantly decreased expression (|log_2_FC| > 0.58, *q* < 0.05) in the VIG group (Fig. 9A and Fig. S8A). According to the KEGG enrichment analysis, ECM–receptor interactions and adherens junctions may have significant roles in this process (Fig. 9B). GO analysis revealed a significant difference in the functional pathways of FAs (focal adhesion) between the 2 groups (Fig. S8B). Moreover, these specific functional pathways were enriched in the VIG group during the GSEA (Gene Set Enrichment Analysis) (Fig. S8C). Combined with RNA-seq analysis, we identified a gene that may contribute to viscoelastic load-induced degeneration of NP cells. Yap1 expression, which is believed to play a crucial role in mechanical stress transmission, was significantly up-regulated in the VIG group, regardless of the presence or absence of viscoelastic loading. RT-PCR analysis showed up-regulation of Yap1 expression in the VIG group of cells, which was further supported by sequencing results (Fig. 9C to E). Furthermore, protein level detection revealed a significant 50% increase in Yap1 protein levels in the VIG group compared to that in the IL-1β group (Fig. S8D and E). These results suggest the potential initiation of the YAP/TAZ signaling pathway after VIG intervention, which may be attributed to the stiffness and viscoelastic properties of the hydrogel material, eliciting a response from the YAP protein [39]. KEGG analysis showed ECM–receptor interactions, indicating the activation of signaling pathways that lead to the expression of ECM in response to YAP proteins. Integrins play crucial roles as key proteins therein [40]. These findings suggest that rapid stress relaxation may stimulate contraction and remodeling of the ECM of NP cells, thereby facilitating enhanced intracellular translocation of integrins and YAP within the cells.

The differential expression of the ECM–receptor interaction pathway suggested that integrin β3 might play a pivotal role in the mechanical response process [41]. Western blotting and RT-PCR results indicated a decrease in integrin β3 expression in the VIG group. Moreover, immunofluorescence analysis revealed a decrease in integrin β3 expression in the VIG group (Fig. 9F to H). Therefore, NP cells regulated the expression and subcellular localization of intracellular Yap1 proteins in response to viscoelastic loading through a mechanotransduction pathway involving integrin β3. This might modulate ECM synthesis and organization of the ECM.

## Conclusion

A VIG with high injectability, frequency-dependent viscoelasticity, suitable degradation rate, NP-biomimetic swelling behavior, and ionic bioactivity was created using PVA and magnesium ions. The application of VIG effectively restores the high viscoelasticity of NP and may potentially facilitate medullary regeneration through specific mechanisms. The VIG treatment effectively restores the biomechanical function of IDD and modulates the local inflammatory microenvironment to mitigate disc degeneration. In summary, we propose a simple and curative strategy to treat IDD and believe that VIG could serve as a promising candidate for minimally invasive IDD treatment.

## Data Availability

The data are available from the corresponding author on reasonable request.

## Supplementary Materials

Supplementary 1 Figs. S1 to S8 Ethics
