# Supplementary material for: Viscoelastic Hydrogel Promotes Disc Mechanical Homeostasis Repair and Delays Intervertebral Disc Degeneration via the Yes-Associated Protein Pathway
Source: Biomater Res. 2025 Mar 4;29:0150. doi: 10.34133/bmr.0150 (PMC11876543; doi:10.34133/bmr.0150)

**Supplementary Materials**


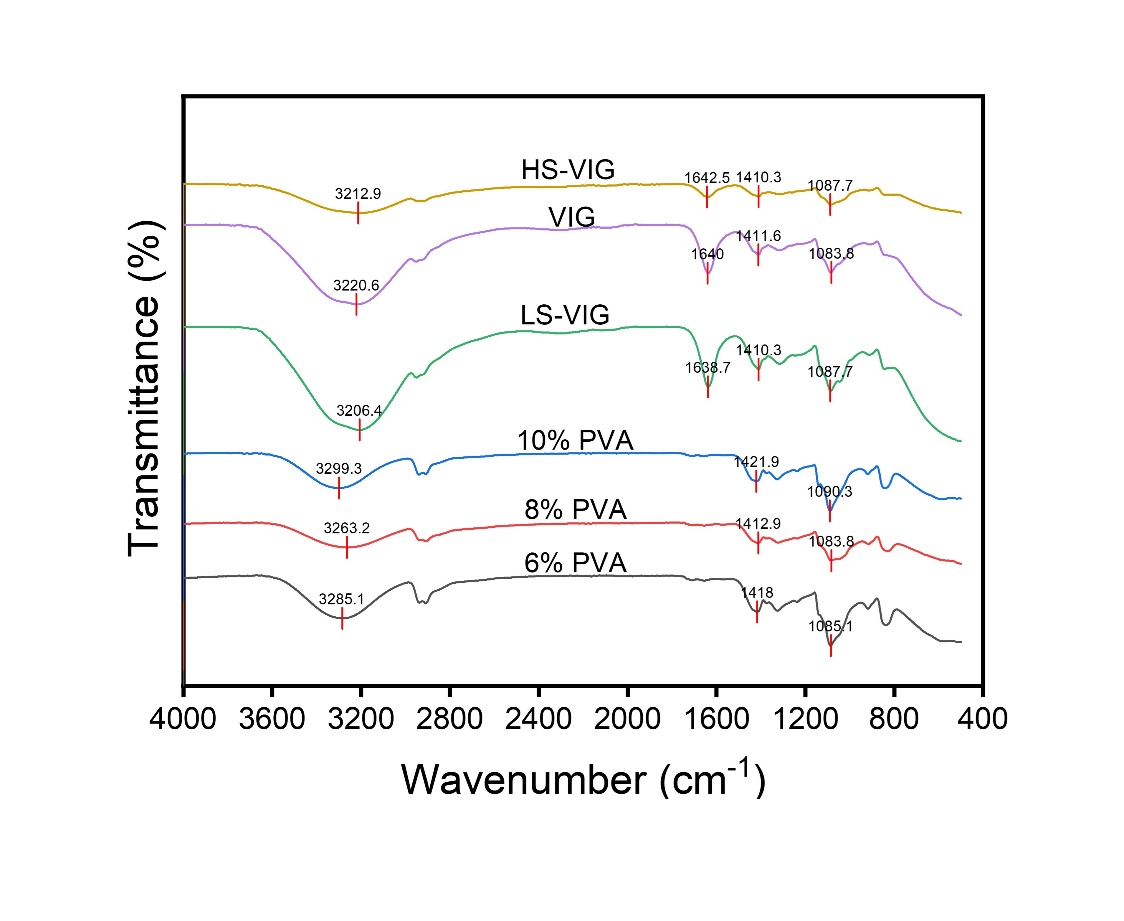


**Figure S1.** FTIR spectrum of different hydrogels.


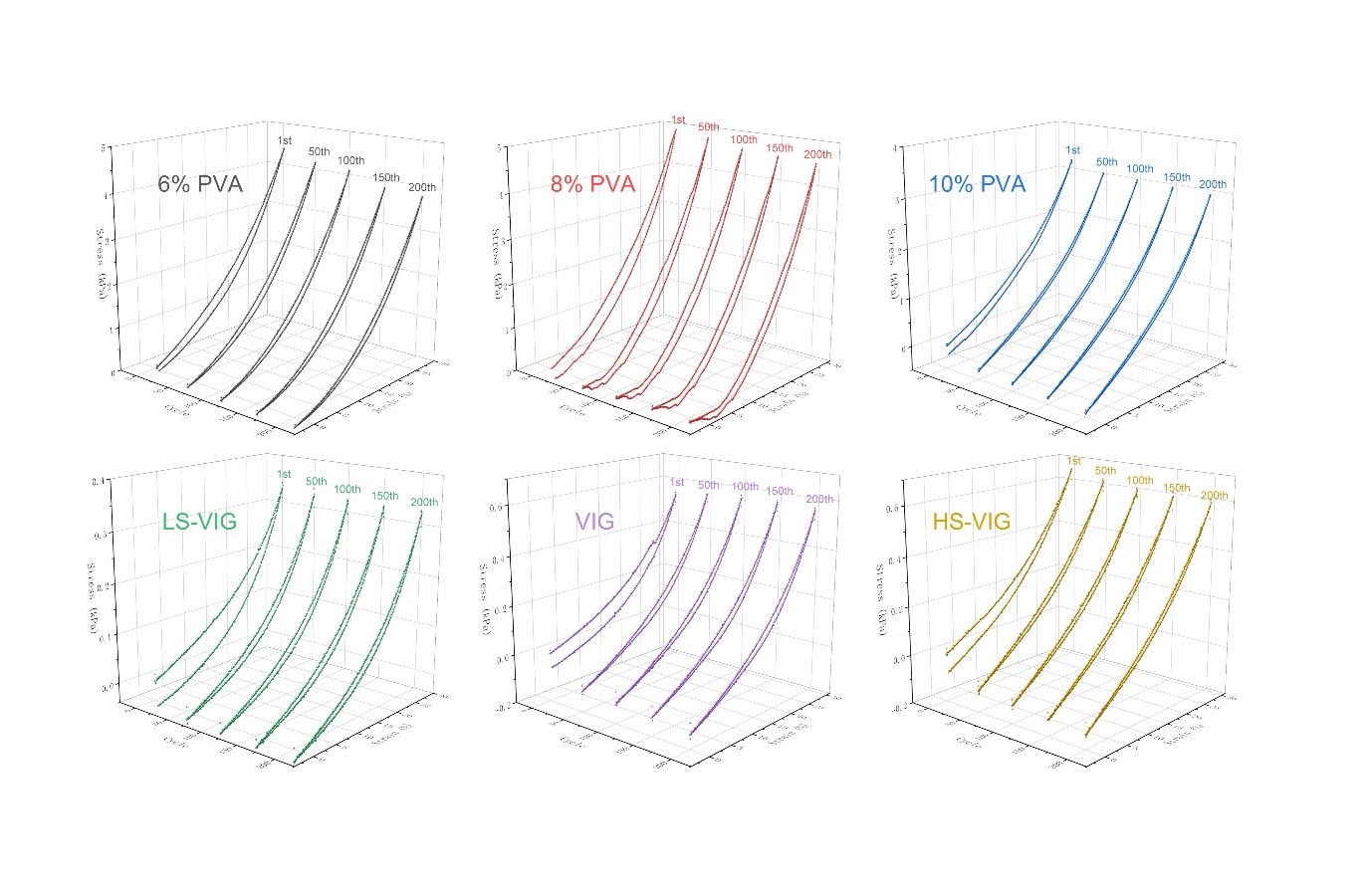


**Figure S2.** Stress–strain curves of cyclic compressive loading-unloading tests on different hydrogels.


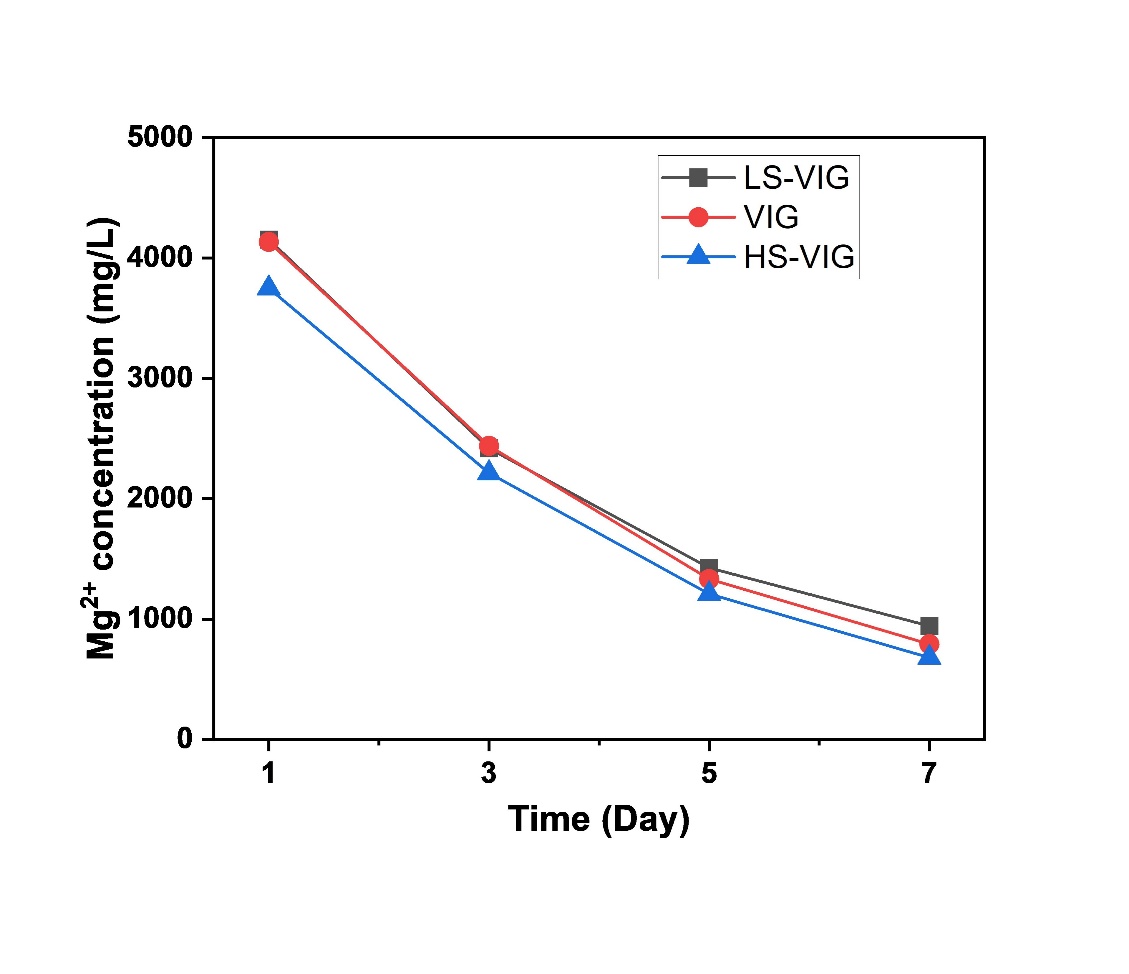


**Figure S3.** The release profile of Mg^2+^ from VIGs.


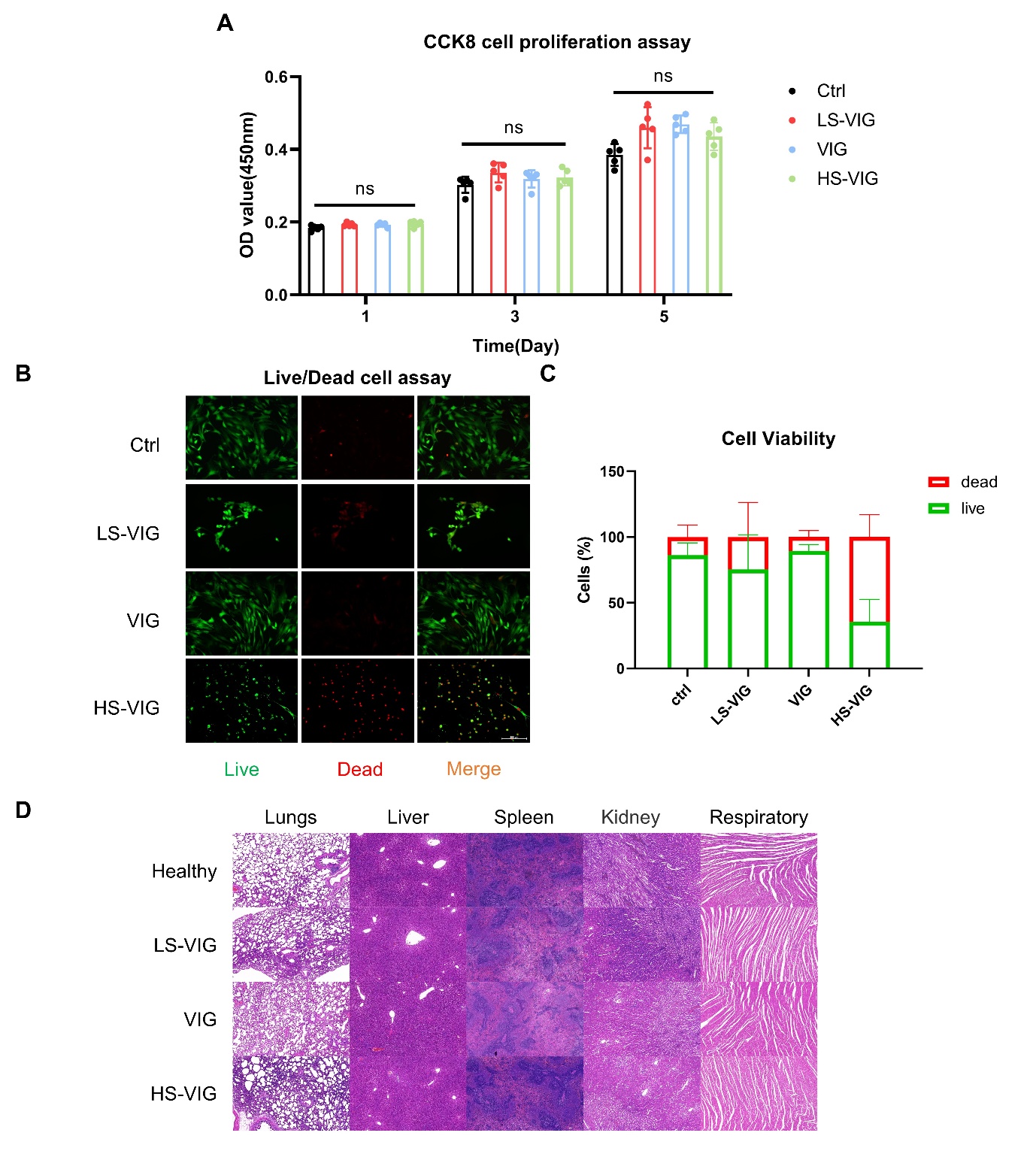


**Figure S4.** Biocompatibility characterization of VIGs. A) CCK8 proliferation assay of NP cells cultured in VIGs extract. B) Staining of live and dead cells after 24 hours of co-culture of VIGs with NP cells. C) Quantitative analysis of live-dead cell staining. D) H&E staining of organ tissues of respiratory, liver, spleen, lungs and kidneys of healthy rats and those treated with 6% VIG, 8% VIG and 10% VIG. Scale = 200μm. *: *P*<0.05, **: *P*<0.01, and ***: *P*<0.001.


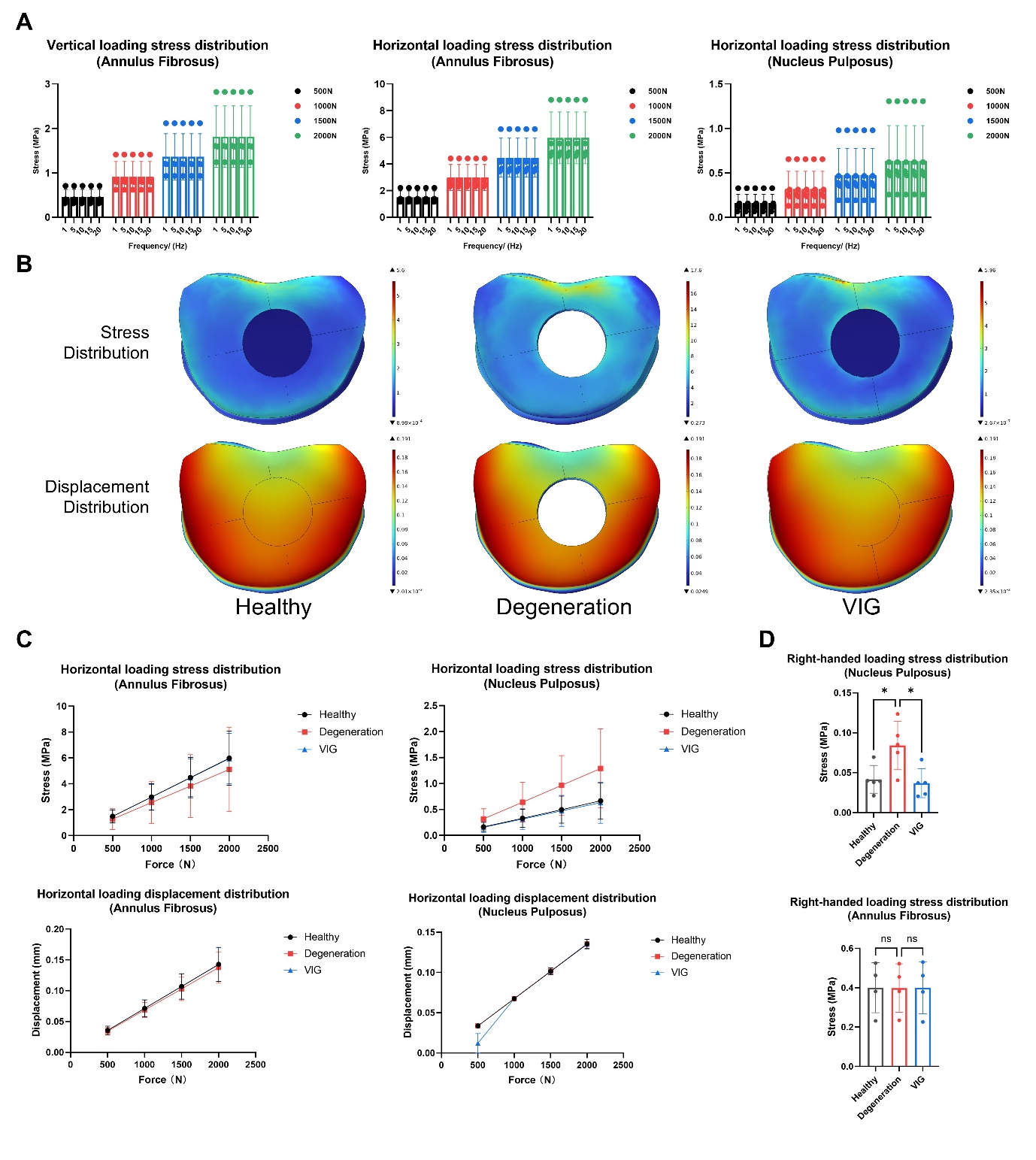


**Figure S5.** Mechanical characterization of VIG simulations for in vivo finite element analysis. A) Average stress distribution on the NP and annulus fibrosus at different frequencies of horizontal loading and on the annulus fibrosus at different frequencies of vertical loading. B) Distribution of intervertebral disc stress and displacements in healthy, degenerative and VIG treatment groups under horizontal loading. C) Quantitative analysis of the distribution of stress and displacements in NP and annulus fibrosus under horizontal loading. D) Quantitative analysis of the stress distribution in NP and annulus fibrosus under right-handed loading. *: *P*<0.05, **: *P*<0.01, and ***: *P*<0.001.


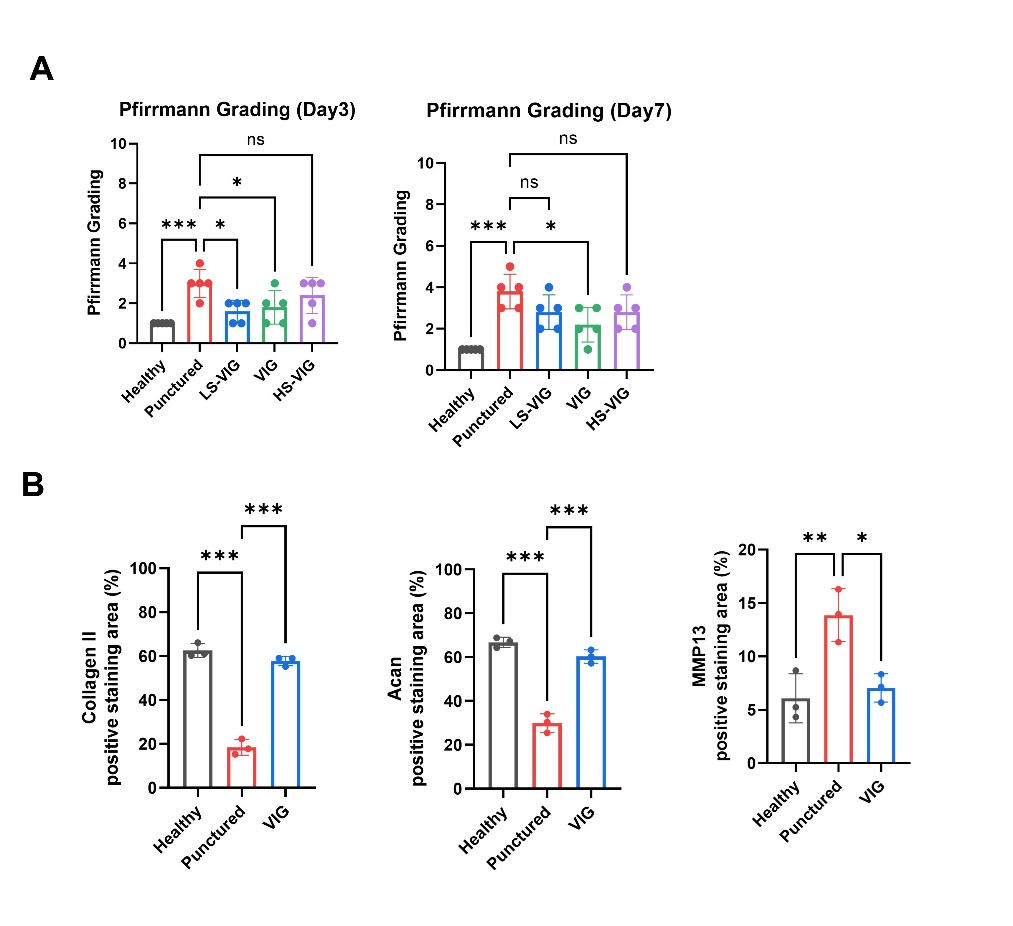


**Figure S6.** Radiological scoring and quantitative histological analysis of a rat model of IDD degeneration. A) MRI index of IDD models. B) Cumulative histological scores at 7 day postoperatively. *: *P*<0.05, **: *P*<0.01, and ***: *P*<0.001.


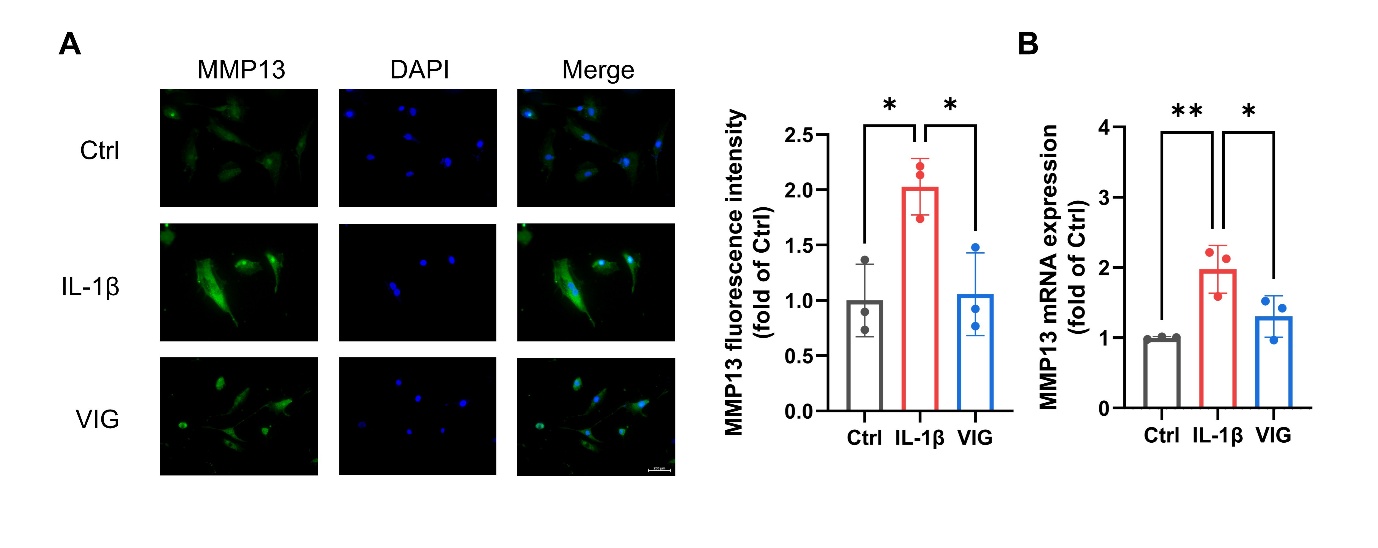


**Figure S7.** Characterization of NP extracellular matrix metabolism. A) Fluorescent staining and semi-quantitative analysis of MMP13 proteins in NP cells. B) RT-PCR results of relative mRNA levels of MMP13 in NP cells after treatment with VIG. Scale = 100μm. *: *P*<0.05, **: *P*<0.01, and ***: *P*<0.001.


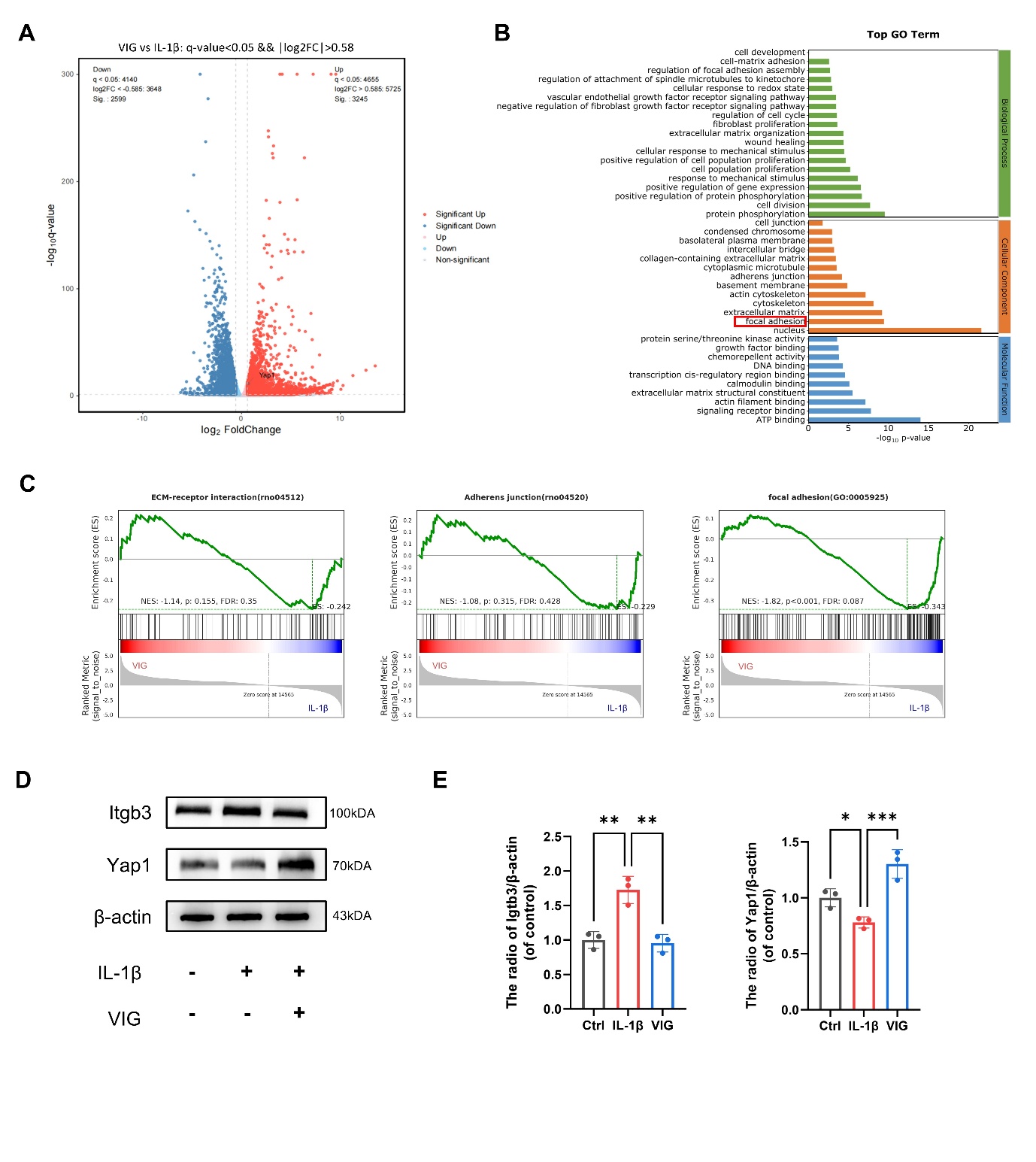


**Figure S8.** Transcriptome sequencing and protein determination in NP cells. A) Volcano plots of differentially expressed genes in NP cells treated with VIG for IL-1β intervention. B) GO enrichment analysis of differentially expressed genes. C) ECM-receptor interaction, Adherens junction and focal adhesion from GESA results comparing IL-1β vs VIC. D) Itgb3 and Yap1 proteins were measured by Western blotting in NP cells treated with VIG for 24 hours. E) Quantitative analysis of Itgb3 and Yap1 proteins detected by Western blotting. *: *P*<0.05, **: *P*<0.01, and ***: *P*<0.001.

**Ethics**


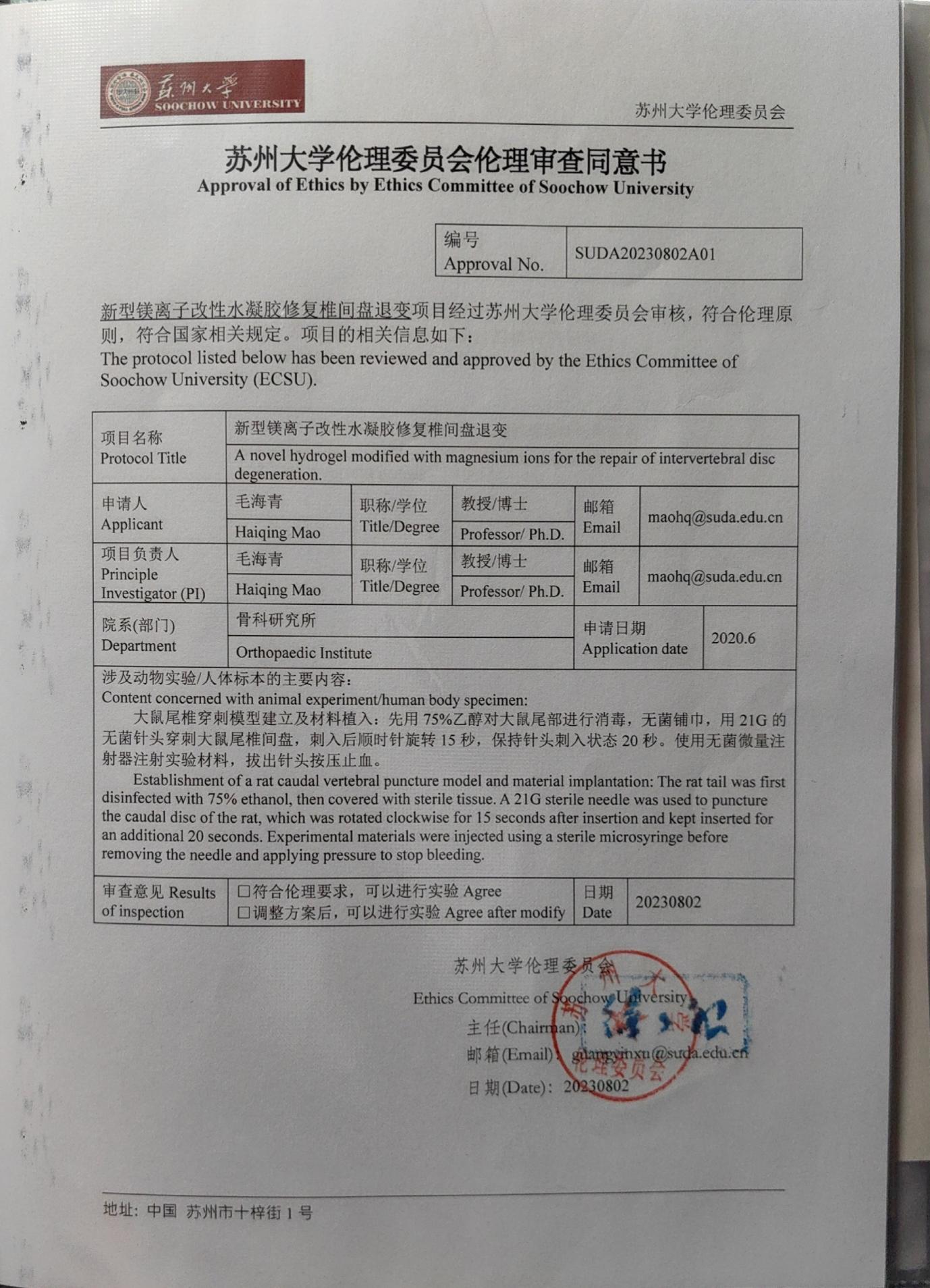

Supplement: Supplementary 1 — Figs. S1 to S8 Ethics [file bmr.0150.f1.docx]
